# Importance of temporary and permanent snow for new second homes

**DOI:** 10.1007/s00484-022-02420-0

**Published:** 2023-01-06

**Authors:** Martin Thomas Falk, Eva Hagsten, Xiang Lin

**Affiliations:** 1 University of South-Eastern Norway, Bø, Norway; 2 Södertörn University, Huddinge, Sweden

**Keywords:** Second homes, Glaciers, Snow depth, Natural amenities, Spatial econometric models, Panel count data models, Norway

## Abstract

This study investigates empirically how natural snow depth and permanent snow affect the number of new second homes in Norway. One out of four Norwegian municipalities is partly covered by glaciers and permanent snow. In the winter seasons of 1983–2020, there is a decline in snow depth from 50 to 35 cm on average (based on 41 popular second-home areas in the mountains). Results of the fixed effects Poisson estimator with spatial elements show that there is a significant and positive relationship between natural snow depth in the municipality and the number of second homes started. There is also a significant and negative relationship between the number of new second homes in the municipality and a scarcity of snow in the surrounding municipalities. However, the magnitude of both effects is small. Estimates also show a strong positive relationship between the proportion of surface covered by permanent snow or glaciers in the municipality and new second homes. This implies that a decline in permanent snow and glaciers may make these areas less attractive for the location of second homes.

## Introduction

In the Nordic countries, the tradition of second homes is both an important form of domestic tourism and a part of the cultural heritage (Ellingsen and Hidle 2013; Müller 2021). Despite this, these homes are rarely included in analyses of tourism demand linked to weather and climate variability (Steiger et al. 2019, Steiger et al. 2022 for literature reviews; Scott et al. 2020 for the Norwegian alpine ski industry). The second homes are commonly located in typical areas for summer or winter recreation, or closer to home as weekend retreats (Slätmo and Kristensen 2021). There is an ongoing discussion about the relationship between tourism demand and climate, particularly so in relation to winter sports destinations and snow (Steiger et al. 2019, 2022). Recent projections show that the snow depth in the Northern Hemisphere will decrease by more than 20% in the winter months towards the end of the century (Zhu et al. 2021). Morin et al. (2021) predict that the number of days with more than 30 cm of natural or artificial snow at an altitude of 800 m in the Norwegian mountains will decrease by 50 to 100 days until the end of the century.

Regardless of the presumptive importance of natural snow conditions for the settlement of new second homes, this is a subject not yet investigated in the literature, although there are several studies on the relationship between house prices and snow conditions (Butsic et al. 2011; Galinato and Tantihkarnchana 2018; Gourley 2021). Butsic et al. (2011) use the hedonic price method to estimate the impact of the percentage of precipitation falling as snow over the winter months on house prices and find a decrease near ski resorts in the Rocky Mountains with lower snowfall. Another examination of snowfall and house prices in the United States demonstrates a positive and significant relationship in the Midwest and Northeast regions based on a spatial autoregressive model (Galinato and Tantihkarnchana 2018). Gourley (2021) employs the fixed effects model and demonstrates that snow conditions could affect the attractiveness of a property in either direction, but there is no significant time delay in the reaction. The COVID-19 pandemic period exhibits both a rising demand for second homes and a growing need for or interest in research within this field (Alonsopérez et al. 2022; Zoğal et al. 2022). Adie (2020) points out the vulnerability of second homeowners to natural hazards, which are likely to increase due to climate change. There is even evidence that second homes are less prone to climate change–related risks than other property owners (Rey-Valette et al. 2015).

In this study, the determinants of demand for new “second homes” between 1983 and 2021 are investigated. A specific focus is put on the role of snow depth as well as on glaciers and permanent snow within the area or in its surroundings. Data employed for the study encompass the number of second homes started in 354 municipalities in Norway. A subsample of 41 municipalities with dense clusters of second homes where information on snow depth is also available is used for a more in-depth analysis. The conditional fixed effects Poisson estimator is applied. Official data on snow depth from the Norwegian centre of Climate Service and detailed land use characteristics from Statistics Norway are employed to guarantee consistency in quality and measurement over the period of time studied.

Presently, the construction of between 6000 and 8000 second homes starts in Norway every year. This is a substantial development since the early 1980s with the building of approximately 2000 second homes per year (source: Statistics Norway). A considerable part of these new properties is located in the mountains (source: Statistics Norway). However, from the mid-2000s to the mid-2010s, the growth momentum of second homes in the mountains is slowing down compared with other areas (Fig. 2, Appendix). The increasing number of winter seasons with a lack of snow may lie behind this. In Norway, the natural snow depth decreases from 50 to 35 cm on average during the winter months in the period 1983–2020 (December to April) (based on 41 second home areas in the Norwegian mountains; Source: Norwegian centre for Climate Services). An absence of natural snow could stimulate interest in locations closer to areas with permanent snow. In Norway, one out of four municipalities has glaciers or permanent snow in the vicinity (source: Statistics Norway).

A major contribution of this study is the use of data on snow depth, permanent snow, glaciers and nature-based amenities as explanations for the development of new second homes. The spatial effects and the characteristics of the dependent variable as a count are taken into account. An advantage of the panel data model is that it controls for presumptive measurement errors that are time-invariant, such as distances to large agglomeration areas. Several studies indicate that glaciers are an important attraction factor for visitors and tourists (Welling et al. 2015; Salim et al. 2022), something that could influence the choice of location for new second homes. Yuheng et al. (2022) use a spatial econometric count data model to estimate the determinants of the number of second homes at the detailed county level. Their estimates show a relationship with natural amenities. The present study extends this line of work with panel data count models augmented by spatial effects and the inclusion of snow depth. Information on land use characteristics are also more detailed than in previous studies (Willibald et al. 2019; Yuheng et al. 2022).

What is more, the present analysis contributes to the literature on determinants of second homes based on regional data (De La Mata and Llano-Verduras 2012; Yuheng et al. 2022). For example, Yuheng et al. (2022) model the determinants of the share of second homes using spatial econometric models. The results based on 360 counties in Corsica show that the proportion of second homes depends on the physical landscape as well as the presence of lakes, a coastline or mountains 500 m or higher. De la Mata and Llano-Verduras (2012) study the determinants of second home stays using a gravity model and find that the number of trips depends on the distance, the added value of the hotel sector and the GDP in the regions.

The study is structured as follows: This introduction is followed by sections on the conceptual background, empirical approach, data, results and conclusions.

## Conceptual background and previous literature

The concept of domestic tourism is not limited to stays in hotels or other accommodations but also includes second homes in a broader sense (Müller 2002; 2021). Second-home properties are unevenly distributed geographically and the change over time in numbers is slow (Back and Marjavaara 2017; Barke 2007; Mowl et al. 2020; Müller 2021).

The empirical model is based on the theory of demand for housing or second homes, where the number of these properties is a function of income and prices as well as a group of wider factors including destination and landscape attributes such as the general infrastructure, natural and cultural resources, recreation and sports facilities as well as accessibility (Mayo 1981; Yuheng et al. 2022). Literature also demonstrates that the demand for second homes depends on socio-economic characteristics of the owner (Skak and Bloze 2017).

Individual or household income is a crucial factor behind the purchase or building of a second home (Skak and Bloze 2017). Hall and Müller (2004) argue that buying a second home could be part of a personal investment strategy, especially for people with middle or high incomes. The present study employs aggregate real GDP to control for income. In addition to income, the affordability of housing is considered an important factor (Yuheng et al. 2022). In the empirical part of the study, the affordability of housing is measured by the construction price index.

In many areas of Norway, the snow season is long and winter sports such as cross-country skiing are popular leisure activities that 40% of the population engage in at least once a year (Sælen and Ericson 2013). The role of natural snow for downhill skiing based on historical data or based on climate scenarios is well studied (Steiger et al. 2019 for an overview). Research that models the relationship between winter tourism demand and snow conditions uses data with different frequencies (at the daily level see Englin and Moeltner 2004; Parthum and Christensen 2022; at the monthly or annual data see Prettenthaler et al. 2022), levels of aggregation (ski resorts, villages and regions) or definitions of winter tourism demand (skier visits, lift ticket sales, overnight stays, arrivals).

Natural snow depth is commonly used as an indicator of snow conditions. Several alternative measures are proposed in the literature, such as the snow reliability index, defined as the proportion of the surface of the gravitational envelope with a minimum amount of snow for skiing (Berard-Chenu et al. 2021). An alternative measure is the number of days with at least × centimetres of snow on the ground or the snowmaking potential, that is, the number of hours with a wet-bulb temperature lower than that required for producing snow (Abegg et al. 2021). Based on the availability of data, the average natural snow depth for the winter season is employed.

For analyses of the impact of climate change on winter tourism demand in the form of second homes, data over longer periods of time are required. Martín (2005) points out that the frequency of data is crucial in estimating the impact of weather on tourism. Over a longer period of time, snow conditions tend to have less of an impact on tourism flows, as visitors can change to periods of good snow conditions (intertemporal substitution). Besides this, there is also a possibility to relocate to areas nearby with better snow conditions (Falk et al. 2022). This implies that there can be spatial effects of snow conditions in the surrounding area. As opposed to general tourism demand, the decision to buy or extend a second home involves longer decision-making, planning and construction processes and costs, implying that possible relationships may appear asynchronously.

Studies using annual data for the whole winter season at the regional or village level show a significant but not always positive relationship between snow depth and overnight stays or arrivals (Töglhofer et al. 2011; Damm et al. 2017; Falk and Lin 2021; Prettenthaler et al. 2022). However, the relationship is small and decreases over time (Falk and Lin 2021). Damm et al. (2017) provide estimates on the snow sensitivity of overnight stays in the winter season based on NUTS3 data from twelve European countries in the period 2005 to 2010. In about half of the 119 regions, there is a significant positive relationship between snow conditions and overnight stays. Given that the demand for second homes is sensitive to several attributes such as natural resources (Mayo 1981; Yuheng et al. 2022), natural snow is expected to be one of these aspects, just like the demand for general tourism to winter sports regions. These considerations lead to the first set of hypotheses:

- H1a: Municipalities with reliable snow conditions within the area or close by are more attractive for the establishment of new second homes.
- H1b: The relationship between natural snow and the establishment of new second homes is asynchronous.
  Another important factor in the demand for outdoor recreation is the landscape (Hansen 2021; Kienast et al. 2012; Willibald et al. 2019). Willibald et al. (2019) examine the attractiveness of landscapes for outdoor recreation, paying particular attention to accessibility, ruggedness, infrastructure for outdoor activities (hiking, biking, skiing), streams and rivers, lakes, forests as well as alternative land use/cover. Other studies that investigate the demand for outdoor recreation show the importance of natural areas such as national parks (Schägner et al. 2017) and forests (Agimass et al. 2018). In this study, seven different characteristics of the landscape are used of which permanent snow and glaciers are the determinants in focus.
  A related literature investigates the relationship between second-home property prices and amenities. For instance, Nilsson (2015) finds that natural amenities significantly increase second-home prices in rural areas in Sweden. Demiroglu et al. (2023) show that second homes are indeed concentrated to exposed locations such as mountain, river and coastal regions, which have a significant potential climate change-related risk due to an increase in forest fires, floods and decreasing snow reliability while Naldi et al. (2021) document that natural amenities are an important location factor for new firms in Sweden.
  The literature on the demand for second homes demonstrates that buyers or builders of these properties seek a home near nature or in amenity-rich landscapes such as mountain, coastal, lake and river areas (Müller 2002; Kaltenborn et al. 2008; Norris and Winston 2009; Yuheng et al. 2022). Marjavaara and Müller (2007) suggest that second homes are commonly located near lakes, coasts and mountains, close to nature with many outdoor recreational opportunities and amenities or in remote areas. The location of second homes is likely to be higher in amenity-rich landscapes in the surrounding area, suggesting that spatial effects need to be considered (Yuheng et al. 2022). Thus, the second hypothesis is formulated:
- H2: Municipalities with natural attractions or recreational facilities within the area or close by are more attractive for the establishment of second homes.
  Another factor for the location of second homes is the presence of glaciers or areas with permanent snow. These are major attractions for visitors and offer opportunities for skiing, adventure tours, hiking or mountaineering (Furunes and Mykletun 2012; Welling et al. 2015; Salim et al. 2022). Several studies find that the most important motivations of glacier visitors are the closeness to nature, seeing its beauty and discovering new things (Welling et al. 2015; Salim et al. 2022). These factors could also be relevant for the settlement of new second homes. However, glacier retreats may lead to a decline in the attractiveness of the area for visitors (Welling et al. 2015; Salim et al. 2022). The presence of glaciers is also an indicator of altitude and snow reliability. It can be assumed that in times of global warming, these areas are preferred locations for second home builders as they offer the possibility of snow-based winter sports activities. Several studies on downhill ski tourism show that visitors move to other locations or change kinds of activities in winters with little snow (Dawson et al. 2011; Cocolas et al. 2016) and that higher altitude areas benefit from such winters (Steiger 2011). The same reasoning might be applied to the demand for second homes, although this change is expected to take longer time. Willibald et al. (2021) show that winter sports activities at medium altitudes are susceptible to climate variability, while winter sports resorts at low altitudes cannot meet certain thresholds for snow reliability. In contrast, snow reliability in high-altitude winter sports resorts is always guaranteed. The emphasis on high altitudes means that areas with access to glaciers or permanent snow can offer snow security and are therefore preferred locations for new second homes. This leads to the third hypothesis:
- H3: Municipalities with permanent snow or a glacier within the area or close by are attractive for the establishment of second homes.
  Accessibility and geographical distance from the main residence are also important aspects for the location of a second home (de la Mata and Llano‐Verduras 2012). However, even if individuals move homes from time to time, this does not happen each year, implying that the variable has features that cannot explain the change in demand for new second homes over time. Another factor affecting housing construction is legal constraints such as land availability and policy limitations (permit fees, restricted density policies, building permits, etc.) (Yuheng et al. 2022; Stricker 2022). Unfortunately, no detailed information is available on this over time.

## Empirical model

In the following, it is assumed that the number of second homes at the municipality level depends on income, prices, snow conditions and a set of land use characteristics (Willibald et al. 2019; Yuheng et al. 2022). Since demand for second homes is not only influenced by characteristics within the area, but probably also by its broader surroundings, an SLX model is used where the independent variables are spatially lagged (Elhorst 2014; Yuheng et al. 2022). Time-invariant characteristics such as the presence of lakes or distance to agglomerations are factors captured by the municipality fixed effects. Two datasets are employed: The first is a longer panel that includes information on snow depth for a group of municipalities in mountainous areas, the shorter panel dataset relates the number of second homes started to land use characteristics across all municipalities.

In the first empirical specification (Eq. 1), the number of second homes started, $$Secondhomes$$, in a given municipality (*i*) with *i* = 1,∙ ∙ ∙, 41 and year (*t* = 1983*,∙ ∙ ∙,* 2021), is related to the natural snow depth from December to April measured as the mean in logarithmic form (*lnSnowdepth*) and to the spatially weighted versions of snow depth ($$W\cdot lnSnowdepth)$$. The latter accounts for possible regional spillover effects or dependencies. In addition, the demand equation contains the gross domestic product in constant prices ($$GDP)$$ and the construction price index (*PI****)*** as income and price variables*.* Other time-varying factors are captured by dummy variables for different decades. Thus, the demand for new second homes is specified as:

1 $${Secondhomes}_{it}={\alpha }_{i}+{{\alpha }_{1}lnGDP}_{t-1}+{\alpha }_{2}{lnPI}_{t-1}+\sum\limits_{j=1}^{3}{\beta }_{j}{lnSnowdepth}_{it-j}+\sum\limits_{j=1}^{3}{\gamma }_{j}W\cdot {lnSnowdepth}_{it-j} +{\alpha }_{3}d1990\_1999+{\alpha }_{4}d2000\_2009+{\alpha }_{5}d2010\_2019+{{\alpha }_{6}d2020\_2021+\varepsilon }_{it}$$

where ln() denotes the natural logarithm and *W* is a N*N spatial weight matrix based on the inversed geographical distance between the municipalities. This is normalised by dividing each element with the modulus of the largest eigenvalue of the matrix (spectral normalisation), $\varepsilon$ is the error term and ${\alpha }_{i}$ represents the municipality fixed effects. The snow depth variables are lagged up to 3 years to allow for time-delayed response and measures the average of the whole winter season (December in year *t* − 1 to April in year *t*), while the number of new second homes refers to the status at the end of the year *t*. This means that there is a slight time lag between these two variables already in the contemporaneous relationship. Dummy variables, *d*s, are associated with a set of time periods.

In the second specification (Eq. 2), the number of second homes started is related to land use characteristics and control variables:

2 $${Secondhomes}_{it}={\alpha }_{i}+{Landuse}_{it}{{\widetilde{\mathrm{Z}}}_{1}+{W\cdot Landuse}_{it}{\widetilde{\mathrm{Z}}}_{2}+{\alpha }_{T}T+\widetilde{\varepsilon }}_{it}$$

where *i* represents the municipalities with *i* = 1,∙ ∙ ∙, 354 and year (*t* = 2013*,∙ ∙ ∙,* 2021). The vector of land use characteristics *Landuse* consists of bare rock, gravel and blockfields, permanent snow and glaciers, recreational facilities, green areas and sports facilities, forest, inland water and wetland, all measured as the proportion of surface to the total land surface. A similar spatial matrix is used as in Eq. 1, although in this case together with the land use characteristics ($W\cdot Landuse).$ Time effects, *T*, are included to capture macroeconomic movements such as economic growth and inflation over a short period of time.

The two equations are estimated using the SLX model in which only local spatial effects are considered. Vega and Elhorst (2015) point out that the SLX is a good starting point when there are no clear theoretical guidelines. The number of second homes started is an integer with a certain proportion of zero values (4% in the long panel dataset and 11% in the shorter) and a distribution skewed to the right where the median of the second homes is far smaller than the mean (long sample 21 to 35 and short sample 9 to 19) (Fig. 5, Appendix). Therefore, the fixed effects Poisson maximum likelihood estimator with cluster-adjusted standard errors at the municipality level is employed. Wooldridge (1999) suggests that this estimator is more favourable for panel count data than the fixed effects negative binomial estimator. Parameter estimates and their robust (sandwich) variance are consistent even if the data are not Poisson-distributed (Wooldridge 1999; Gourieroux et al. 1984).

It can be assumed that the causality goes from snow to the demand for new second homes, since temporary or permanent snow is exogenous in the short and medium term. This is not always a correct assumption—for the other variables. Velvin et al. (2013) expect that second-home owners demand a high standard of infrastructure and various other facilities in the region. This shows that there is a possible reciprocal relationship between new second homes and leisure as well as sports facilities. Therefore, this relationship should be interpreted with caution.

## Data

Data for this analysis originates from two main sources: Statistics Norway provides information on new second homes started and completed, land use characteristics, GDP and the construction price index while information on snow depth is available at the Norwegian Centre for Climate Services.

The dependent variable “number of second homes started” can be found in the building statistics at Statistics Norway (“Byggeareal Fritidsbygninger”, Holiday or second homes, by region, year and content, number 06952, source: www.ssb.no/en/statbank/table/06952). “Started” in this context means that the building permit is obtained and that the actual construction process is underway (Holst Bloch and Svenkerud 2019). Second homes started also include extensions and buildings with more than one dwelling unit are counted as one. From 2010 onwards, there are definitional changes, buildings with a usable floor space of 16 m^2^ and more are included instead of the earlier measure of 30 m^2^ (Holst Bloch and Svenkerud 2019). The second home data refer to the status at the end of each year.

Land use characteristics originate from the statistics “Land use and land cover (km^2^), by region, area classification, content and year” (number 09594; source https://www.ssb.no/en/statbank/table/09594). There is information on 18 different characteristics for 354 municipalities (Norway has 357 municipalities, excluding Svalbard and Jan Mayen and Continental shelf). Orkland and Hamarøy are excluded from the analysis due to a lack of data for the period 2013 to 2021.

Series of price indexes are based on the construction cost index for residential buildings (2015 = 100; www.ssb.no/en/statbank/table/08651) and GDP in constant prices originates from the national accounts database of Statistics Norway (constant prices with the base year 2015 https://www.ssb.no/en/statbank/table/09842/).

The second data source is the Norwegian Centre for Climate Services (“Meteorologisk institutt—Norsk Klimaservicesenter” https://seklima.met.no). Weather stations selected for the study have long and consistent time series on the mean level of snow depth at the monthly level (Table 7, Appendix). Short gaps are interpolated using the information on neighbouring weather stations. The average monthly snow depth is calculated in centimetres from December year *t − *1 to April year *t*. A total of 61 weather stations in mountain destinations can be assigned to 41 municipalities (Table 6, Appendix). Municipalities in mountains or winter sports areas with a minimum number of four new second homes per year during the time period studied are included in the analysis. These municipalities host 30% of the second homes in Norway. The Norwegian *adm2* shapefile is extracted from the GADM database (www.gadm.org), version 2.5, July 2015, and is used for the creation of the spatial weights.

In Norway, there are approximately 450,000 registered second homes (Statistics Norway). The number of second homes is steadily increasing over time (Fig. 2, Appendix). During the second year of the COVID-19 pandemic (2021), there is a record number of second homes started (8600). Second homes are geographically unevenly distributed across the country (Fig. 3. Appendix). This can be observed based on three indicators (second home stocks, second homes started and second homes completed). Glaciers and permanent snow can be found in 92 out of 354 municipalities and are concentrated to the West and the North of Norway (Fig. 4, Appendix; see also Furunes and Mykletun 2012). Green spaces, sports facilities and recreational facilities are less geographically concentrated and are available across all parts of the country (Fig. 4, Appendix).

**Fig. 1:**
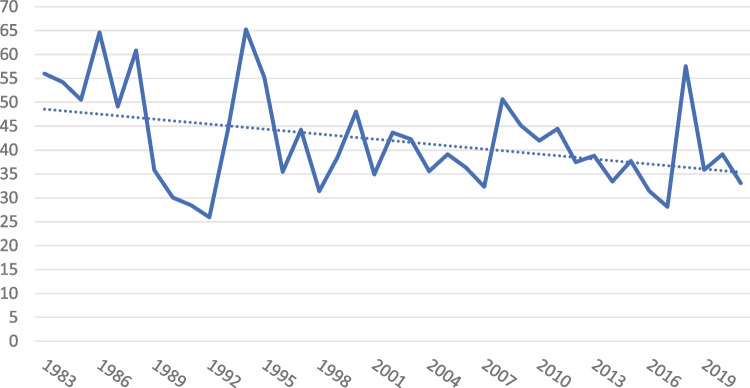
Evolution of snow depth in selected second home areas in Norway (centimetres). Source: The Norwegian Centre for Climate Services

**Fig. 2:**
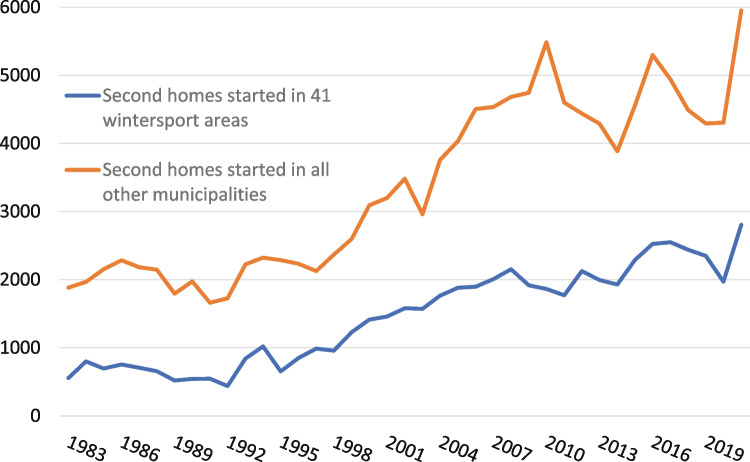
Evolution of the number of second homes started in Norway. Source: Statistics Norway

**Fig. 3:**
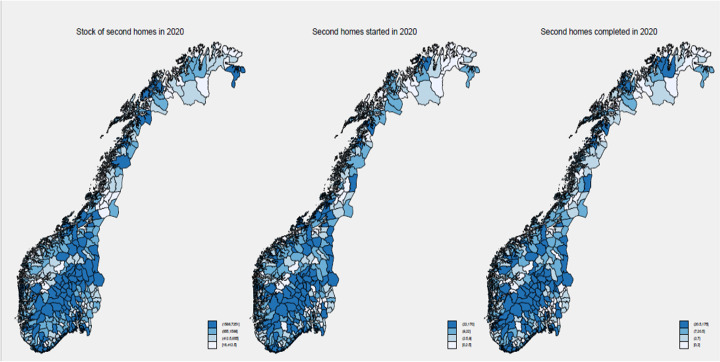
Spatial distribution of the number of second homes in Norway. Source: Statistics Norway. The Norwegian adm2 shapefile is extracted from the GADM database (www.gadm.org), version 2.5, July 2015

**Fig. 4:**
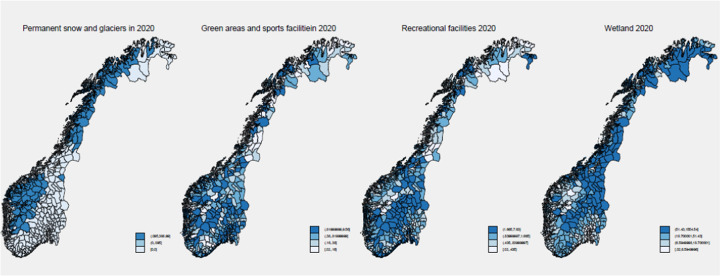
Spatial distribution of selected amenities and landscape characteristics in Norway. Source: Statistics Norway. The Norwegian adm2 shapefile is extracted from the GADM database (www.gadm.org), version 2.5, July 2015

**Fig. 5:**
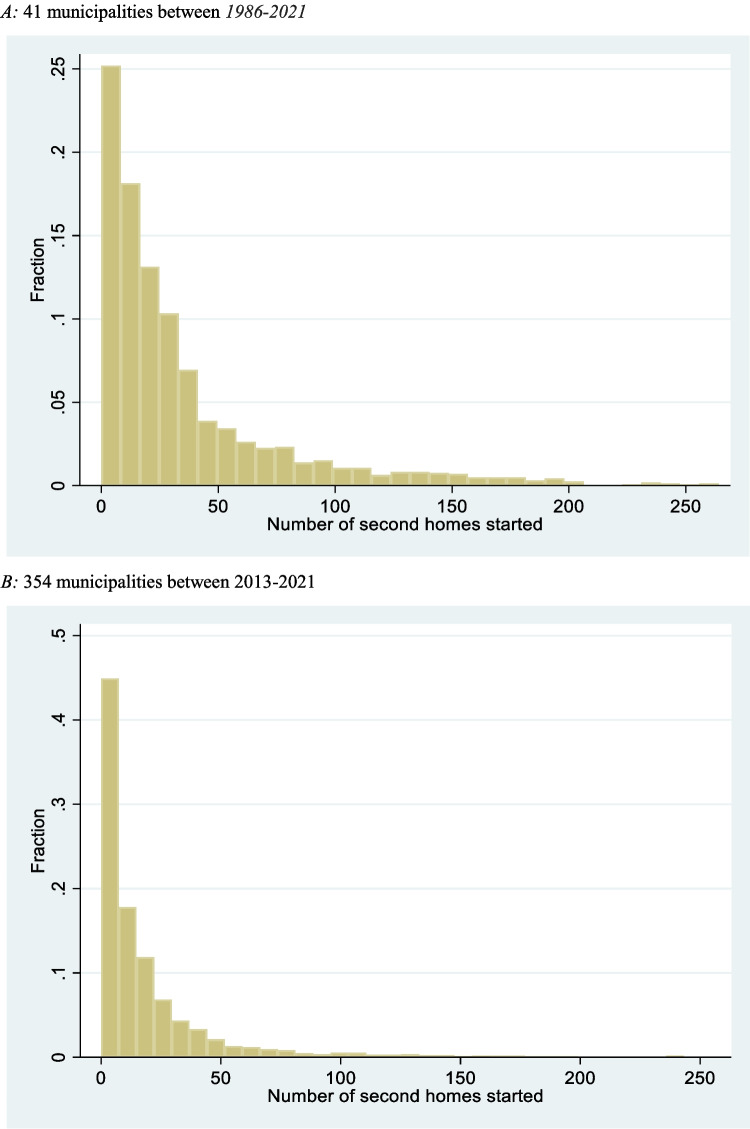
Histograms of the number of second homes started in Norway. **A** 41 municipalities between 1986 and 2021. **B** 354 municipalities between 2013 and 2021. Source: Statistics Norway. Building statistics, www.ssb.no/en/statbank/table/06952

Descriptive statistics also show that natural snow depth decreases from 50 to 35 cm on average during the winters in the period 1983–2020 (December to April) based on the 41 municipalities in the long panel (Fig. 1 and Table 3, Appendix). Simple fixed effects estimations of snow depth on a trend indicate that it decreases on average by 0.35 cm per year, with a *t*-stat of − 8.3. Forests make up the largest part of the municipalities with 41.6%, while bare rocks, graves and boulder fields, wetland and inland waters account for 5.7%, 4.5% and 5.8%, respectively. Other land use features represent a small proportion (permanent snow and glaciers at 0.5%, recreational facilities 0.3% as well as green spaces and sports facilities 0.2%) (Table 4, Appendix).

The proportion of permanent snow and glaciers decreases over time, while the extent of green space and sports facilities, recreational areas, forests and inland water bodies surges. Other land features are stable over time (Table 5, Appendix). When the changes are expressed as proportions, they are small. The most marked alterations are observed for permanent snow and glaciers that decrease by almost 0.2 percentage points over the period of time studied (from 2.67 in 2011 to 2.51 in 2021). Calculated as the level of surface covered by glaciers and permanent snow, the annual decline is on average 1.9% during the years 2011 to 2021.

## Empirical results

Results of the fixed effects Poisson estimator with spatially weighted variables for the long panel data set reveal that there is a significant and positive relationship between snow depth in the previous season and the number of initiated second homes in the municipality when period effects, GDP and construction prices are held constant (Table 1, Models 1–4). The snow depth elasticity is 0.058 and significant at the 5% level. This means that a 50% surge in snow depth the previous winter season (for instance winter 2018/2019) is associated with an increase in the number of second homes started the following year by 2.9% (calculated as 0.058 × 50).

**Table 1:** Determinants of the number of second homes started, fixed effects Poisson estimations (long panel data model)

	Model 1	Model 2	Model 3	Model 4
lag of lnGDP	1.999*** (0.52)	2.365*** (0.55)	2.790*** (0.59)	2.799*** (0.59)
lag of lnPI	0.442 (0.26)	0.237 (0.27)	− 0.070 (0.29)	− 0.077 (0.29)
ln(snow depth)				
1st lag	0.058** (0.03)	0.153*** (0.04)	0.148*** (0.04)	0.147*** (0.04)
2nd lag	− 0.006 (0.03)	− 0.012 (0.03)	0.052 (0.04)	0.051 (0.04)
3rd lag	0.039 (0.03)	0.032 (0.03)	0.031 (0.03)	0.039 (0.03)
Spatial lag W* ln(snow depth)				
1st lag		− 0.250*** (0.06)	− 0.255*** (0.06)	− 0.255*** (0.06)
2nd lag			− 0.190*** (0.06)	− 0.189*** (0.06)
3rd lag				− 0.019 (0.07)
d1990–1999	− 0.189 (0.12)	− 0.287** (0.06)	− 0.337*** (0.13)	− 0.342*** (0.12)
d2000–2009	− 0.009 (0.16)	− 0.113 (0.17)	− 0.163 (0.17)	− 0.167 (0.17)
d2010–2019	− 0.032 (0.18)	− 0.076 (0.19)	− 0.032 (0.19)	− 0.034 (0.19)
d2020–2021	− 0.022 (0.10)	− 0.003 (0.09)	0.077 (0.11)	0.079 (0.11)

Asterisks ***, ** and * denote significance at the 1, 5 and 10% levels. The standard errors are reported within the parentheses. All estimations are based on robust standard errors and conducted using the Stata command xtpoisson and the robust option. Spmatrix is used to create the spatial weight matrix and spgenerate to generate the spatially lagged variables.

Source: Statistics Norway, Norwegian Centre for Climate Services and own calculations.

The coefficient of the spatially weighted value of the natural snow depth is significantly negative at the 1% level, implying that the number of new second homes increases in the municipalities when the surroundings experience one or two consecutive years of decreasing snow cover. These results imply that neither Hypothesis 1a nor 1b can be rejected. Economic factors are partially significant. The GDP elasticity ranges from 2 to 2.8, suggesting that the number of second homes grows disproportionately with a rise in income, similar to luxury goods.

Results for the shorter panel data set including land use characteristics show that amenities and natural attractions are an important aspect of the demand for new second homes (Table 2, Model 1). Both permanent snow and glaciers as well as wetlands are positive and significant at the 5% level. Permanent snow and glaciers in the surrounding area are significant at the 5% level and remain so when the spatially weighted variables are added (Table 2, Model 2). The coefficient for the variable permanent snow and glaciers is 0.156, which means that a decrease of one percentage point (for instance from 2.5 to 1.5%) renders a drop in the number of new second homes by 0.2% per year. Thus, the magnitude of this relationship is small.

**Table 2:** Determinants of second homes started, fixed effects Poisson estimations (short panel data model)

	(Model 1)	(Model 2)
Proportion (per cent)		
Bare rock, grave, land block field	0.081 (0.08)	0.062 (0.08)
Permanent snow and glaciers	0.159** (0.08)	0.208** (0.10)
Recreational facilities	0.669 (0.74)	0.374 (0.77)
Green areas and sports facilities	− 0.338 (0.43)	− 0.452 (0.46)
Forest	0.004 (0.07)	− 0.005 (0.06)
Inland waters	− 0.001 (0.14)	0.010 (0.12)
Wetland	0.079** (0.04)	0.007 (0.05)
Spatial lag W* proportion (%)		
Bare rock, grave, land block field		− 0.630 (0.91)
Permanent snow and glaciers		− 2.706 (2.77)
Recreational facilities		12.589*** (5.71)
Green areas and sports facilities		10.971** (4.95)
Forest		− 0.902 (0.65)
Inland waters		0.521 (1.90)
Wetland		0.951 (1.15)
Time dummy variables	2014–2021	2014–2021

Asterisks ***, ** and * denote significance at the 1, 5 and 10% levels. The standard errors are reported within the parentheses. All estimations are based on robust standard errors and conducted using the Stata command *xtpoisson and the robust option. Spmatrix* is used to create the spatial weight matrix and *spgenerate* to generate the spatially lagged variables. The proportions of the land use characteristics are multiplied by 100

Source: Statistics Norway, Norwegian Centre for Climate Services and own calculations

Recreational facilities in the surrounding areas as well as green space and sports facilities nearby are positive and significant at the five or 1% levels, respectively. Bare rocks, gravel and boulder fields, areas covered with forests and inland water within the municipality or in its surroundings are all not significant. Overall, the significant estimates for glaciers could mean that the demand for new second homes is climbing up to higher altitudes, in attempts to adjust to changed snow conditions. Following these results, it is also not possible to reject Hypotheses 2 and 3.

Economic factors at the national level do not play a role in explaining the regional development of new second homes and are thus not included in the final specification.

## Discussion and conclusions

In this study, the relationship between the number of second homes started and the availability of natural snow nearby or typical land use characteristics in different municipalities in Norway is investigated. A particular focus is placed on the role of seasonal and permanent snow. Two different panel data sets are used for the purpose, one longer and one shorter, based on official information from Statistics Norway and the Norwegian Centre for Climate Services. Fixed effects Poisson estimations with spatially weighted variables suggest that snow depth in earlier years is relevant for the settlement of new second homes, but to a small extent. A higher demand can be observed if there is a lack of snow in the surrounding municipalities. One possible explanation for the small magnitude of the relationship could be that buying or building a second home is more of a lifestyle decision that goes beyond aspects such as winters with little snow. A presumptive future development is that overall demand for second homes becomes even more disconnected from snow and climate aspects. Such an evolution is reinforced by the finding that demand for new second homes is higher in areas where the surroundings offer green spaces as well as facilities for recreation and sports. The higher demand for new second homes in areas with permanent snow and glaciers indicates that there are still certain groups with a strong focus on snow supply. This group is expected to adjust to altitudes when needed and if possible. Higher altitudes are often located in protected and remote areas with limited carrying capacity for tourists and buildings.

A few limitations of the study should be noted. Certain municipalities are not included due to a lack of consistent snow depth data. There are also no individual characteristics of the residents available in the dataset at hand. This means, for instance, that the possible importance for demand of the distance between primary and second homes cannot be included in the analyses. Nor can the role of socio-economic aspects be explored. This may need new surveys and thus is left to future research.

Additional research ideas encompass a broader set of weather indicators such as temperatures and a larger set of countries. Another avenue for future work is to include the property tax which is related to the market value of the second home and as such may also be location specific. Future work may also use other indicators of snow depth, for instance, the number of days with more than 30 cm or the length of the ski season. However, this exercise requires new data.

## Data Availability

Data is available upon acceptance.

## Appendix

**Table 3:** Descriptive statistics: long model (data on 41 municipalities from 1986 to 2021)

	Mean	Standard deviation	Minimum	Maximum
Number of second homes started	37	44	0	264
Snow depth in cm	41	26	1.2	149.4
ln(snow_depth)	3.46	0.80	0.18	5.01

Source: Statistics Norway, the Norwegian Centre for Climate Services and own calculations

**Table 4:** Descriptive statistics: short model (data on 354 municipalities from 2013 to 2021)

	Mean	Standard deviation	Minimum	Maximum
Number of second homes started	20	30	0.00	258
Proportion of total surface (per cent)				
Bare rock, grave, land block field	5.67	7.93	0.00	40.32
Permanent snow and glaciers	0.53	2.018	0.00	20.58
Recreational facilities	0.28	0.54	0.00	6.48
Green areas and sports facilities	0.17	0.31	0.00	2.37
Forest	41.60	23.01	0.00	85.77
Inland waters	5.75	3.48	0.22	30.06
Wetland	4.54	4.22	0.00	31.39

Shares are measured by per cent (proportions multiplied by 100)

**Table 5:** Descriptive statistics land use characteristics

	Proportion of total surface (per cent)		Change in percentage points
	Year 2011	Year 2021	
Land use characteristics			
Bare rock, grave, land block field	5.71	5.71	− 0.005
Permanent snow and glaciers	0.71	0.66	− 0.054
Permanent snow and glaciers (*N* = 94)	2.67	2.51	− 0.163
Green areas and sports facilities	0.16	0.18	0.018
Recreational facilities	0.27	0.28	0.013
Forest	41.49	41.55	0.053
Inland waters	5.73	5.78	0.043
Wetland	4.50	4.50	0.002

Descriptive statistics are based on 354 municipalities. For permanent snow and glaciers, the mean values for the sub-sample are also shown

Source: Statistics Norway and the Norwegian Centre for Climate Services

**Table 6:** List of selected municipalities in winter sport areas/mountains

K-1563 Sunndal	K-3405 Lillehammer		K-4228 Sirdal	K-5033 Tydal
K-3006 Kongsberg	K-3407 Gjøvik		K-4611 Etne	K-5037 Levanger
K-3007 Ringerike	K-3411 Ringsaker		K-4618 Ullensvang	K-5038 Verdal
K-3040 Nesbyen	K-3412 Løten		K-4621 Voss	K-5053 Inderøy
K-3041 Gol	K-3421 Trysil		K-5021 Oppdal	K-5054 Indre Fosen
K-3042 Hemsedal	K-3425 Engerdal		K-5025 Røros	K-5058 Åfjord
K-3043 Ål	K-3439 Ringebu		K-5026 Holtålen	K-5060 Nærøysund
K-3044 Hol	K-3441 Gausdal		K-5027 Midtre Gauldal	K-5401 Tromsø
K-3045 Sigdal	K-3453 Øystre Slidre		K-5028 Melhus	K-5403 Alta
K-3046 Krødsherad	K-3825 Vinje		K-5032 Selbu	K-5429 Kvænangen
				K-5436 Porsanger

Source: Statistics Norway

**Table 7:** List of weather stations used to calculate snow depth at the municipality levels

Ål Iii	Fåvang—Tromsnes	Mære	Sirdal—Sinnes
Ångårdsvatnet	Gausdal—Skogli	Mosvik – Trøahaugen	Snåsa—Nagelhus
Aursund	Gausdal—Skogli	Nerskogen Ii	Sokna Ii
Aursund	Geilo	Nesbyen—Skoglund	Soknedal
Beito	Gol—Stake	Nord-odal	Sopnesbukt
Biri	Grimeli I Krødsherad	Nordstraum I Kvænangen	Søre Brekkom
Børselv Ii	Hafsås	Øksendal	Stugudal—Kåsen
Breivoll	Heggeriset—Nordstrand	Ørsjøsetra	Tromsø
Bulken	Hemsedal—Hølto	Øvre Sirdal	Utgård
Buran	Jotkajavre	Øvstedal	Vea
Eggedal Iii	Kongsberg Ii / Iii	Rauland	Vera Ii
Eikemo	Kotsøy	Røldal	Verdal—Reppe
Eksingedal	Liafoss	Røros Lufthavn	Vermundsjøen
Elverum—Fagertun	Linnes	Røsbjørgen	
Eresfjord	Løksmyr		

Source: The Norwegian Centre for Climate Services

## References

[CR1] Abegg B, Morin S, Demiroglu OC, François H, Rothleitner M, Strasser U (2021). Overloaded! Critical revision and a new conceptual approach for snow indicators in ski tourism. Int J Biometeorol.

[CR2] Adie BA (2020). Place attachment and post-disaster decision-making in a second home context: a conceptual framework. Curr Issue Tour.

[CR3] Agimass F, Lundhede T, Panduro TE, Jacobsen JB (2018). The choice of forest site for recreation: a revealed preference analysis using spatial data. Ecosyst Serv.

[CR4] Alonsopérez MJ, Brida JG, Rojas ML (2022). Second homes: a bibliometric analysis and systematic literature review. J Tour Herit Serv Mark (JTHSM).

[CR5] Back A, Marjavaara R (2017). Mapping an invisible population: the uneven geography of second-home tourism. Tour Geogr.

[CR6] Barke M (2007). Second Homes in Spain: an analysis of change at the provincial level, 1981–2001. Geography.

[CR7] Berard-Chenu L, Cognard J, François H, Morin S, George E (2021). Do changes in snow conditions have an impact on snowmaking investments in French Alps ski resorts?. Int J Biometeorol.

[CR8] Butsic V, Hanak E, Valletta RG (2011). Climate change and housing prices: hedonic estimates for ski resorts in western North America. Land Econ.

[CR9] Cocolas N, Walters G, Ruhanen L (2016). Behavioural adaptation to climate change among winter alpine tourists: an analysis of tourist motivations and leisure substitutability. J Sustain Tour.

[CR10] Damm A, Greuell W, Landgren O, Prettenthaler F (2017). Impacts of+ 2 C global warming on winter tourism demand in Europe. Clim Serv.

[CR11] Dawson J, Havitz M, Scott D (2011). Behavioral adaptation of alpine skiers to climate change: examining activity involvement and place loyalty. J Travel Tour Mark.

[CR12] de la Mata T, Llano-Verduras C (2012). Spatial pattern and domestic tourism: an econometric analysis using inter-regional monetary flows by type of journey. Pap Reg Sci.

[CR13] Demiroglu OC, Müller DK, Back A, Lundmark L (2023) Impacts of climate change on Swedish second-home tourism. In: Adie BA, Hall CM (eds) Second Homes and Climate Change. Taylor and Francis. In press

[CR14] Elhorst JP (2014). Spatial econometrics from cross-sectional data to spatial panels.

[CR15] Ellingsen WG, Hidle K (2013). Performing home in mobility: second homes in Norway. Tour Geogr.

[CR16] Englin J, Moeltner K (2004). The value of snowfall to skiers and boarders. Environ Resource Econ.

[CR17] Falk M, Hagsten E, Lin X (2022) Spatial influence on the distribution of downhill skiers in Sweden. Int J Biometeorol 1-11 10.1007/s00484-022-02259-5 10.1007/s00484-022-02259-5 PMC10864428 35357566

[CR18] Falk M, Lin X (2021). Time-varying impact of snow depth on tourism in selected regions. Int J Biometeorol.

[CR19] Furunes T, Mykletun RJ (2012). Frozen adventure at risk? A 7-year follow-up study of Norwegian glacier tourism. Scand J Hosp Tour.

[CR20] Galinato GI, Tantihkarnchana P (2018). The amenity value of climate change across different regions in the United States. Appl Econ.

[CR21] Gourieroux C, Monfort A, Trognon A (1984). Pseudo maximum likelihood methods: theory. Econometrica.

[CR22] Gourley P (2021). Curb appeal: how temporary weather patterns affect house prices. Ann Reg Sci.

[CR23] Hall CM, Müller KD (2004) Tourism, mobility, and second homes: between elite landscape and common ground. Bristol, Blue Ridge Summit, Channel View Publications 15. 10.21832/9781873150825

[CR24] Hansen AS (2021). Understanding recreational landscapes–a review and discussion. Landsc Res.

[CR25] Bloch VVH, Svenkerud S (2019) Registerbasert byggstatistikk. Dokumentasjon og videre arbeid (Register-based building statistics. Documentation and further work). Statistics Norway. https://ssb.brage.unit.no/ssb-xmlui/handle/11250/2625445

[CR26] Kaltenborn BRP, Andersen O, Nellemann C, Bjerke T, Thrane C (2008). Resident attitudes towards mountain second-home tourism development in Norway: the effects of environmental attitudes. J Sustain Tour.

[CR27] Kienast F, Degenhardt B, Weilenmann B, Wäger Y, Buchecker M (2012). GIS-assisted mapping of landscape suitability for nearby recreation. Landsc Urban Plan.

[CR28] Marjavaara R, Müller DK (2007). The development of second homes’ assessed property values in Sweden 1991–2001. Scand J Hosp Tour.

[CR29] Martín MBG (2005). Weather, climate and tourism a geographical perspective. Ann Tour Res.

[CR30] Mayo SK (1981). Theory and estimation in the economics of housing demand. J Urban Econ.

[CR31] Morin S, Samacoïts R, François H, Carmagnola CM, Abegg B, Demiroglu OC, Pons M, Soubeyroux JM, Lafaysse M, Franklin S, Griffiths G, Kite D, Hoppler A, George E, Buontempo C, Almond S, Dubois G, Cauchy A (2021). Pan-European meteorological and snow indicators of climate change impact on ski tourism. Clim Serv.

[CR32] Mowl G, Barke M, King H (2020). Exploring the heterogeneity of second homes and the ‘residual ‘category. J Rural Stud.

[CR33] Müller DK (2002). Second home ownership and sustainable development in Northern Sweden. Tour Hosp Res.

[CR34] Müller DK (2021). 20 years of Nordic second-home tourism research: a review and future research agenda. Scand J Hosp Tour.

[CR35] Naldi L, Nilsson P, Westlund H, Wixe S (2021). Amenities and new firm formation in rural areas. J Rural Stud.

[CR36] Nilsson P (2015). The influence of urban and natural amenities on second home prices. J Housing Built Environ.

[CR37] Norris M, Winston N (2009). Rising second home numbers in rural Ireland: distribution, drivers and implications. Eur Plan Stud.

[CR38] Parthum B, Christensen P (2022). A market for snow: modeling winter recreation patterns under current and future climate. J Environ Econ Manag.

[CR39] Prettenthaler F, Kortschak D, Woess-Gallasch S (2022) Modelling tourists’ responses to climate change and its effects on alpine ski tourism–a comparative approach for European Regions. J Outdoor Recreat Tour 39:100525. 10.1016/j.jort.2022.100525

[CR40] Rey-Valette H, Rulleau B, Hellequin AP, Meur-Ferec C, Flanquar H (2015). Second-home owners and sea-level rise: the case of the Languedoc-Roussillon region (France). J Pol Res Tour Leis Events.

[CR41] Sælen H, Ericson T (2013). The recreational value of different winter conditions in Oslo forests: a choice experiment. J Environ Manage.

[CR42] Salim E, Mayer M, Sacher P, Ravanel L (2022) Visitors’ motivations to engage in glacier tourism in the European Alps: comparison of six sites in France, Switzerland, and Austria. J Sustain Tour. In press. 10.1080/09669582.2022.2044833

[CR43] Schägner JP, Maes J, Brander L, Paracchini ML, Hartje V, Dubois G (2017). Monitoring recreation across European nature areas: a geo-database of visitor counts, a review of literature and a call for a visitor counting reporting standard. J Outdoor Recreat Tour.

[CR44] Scott D, Steiger R, Dannevig H, Aal C (2020). Climate change and the future of the Norwegian alpine ski industry. Curr Issue Tour.

[CR45] Skak M, Bloze G (2017). Owning and letting of second homes: what are the drivers? Insights from Denmark. J Housing Built Environ.

[CR46] Slätmo E, Kristensen I (2021) Urban–rural linkages: an inquiry into second-home tourism in the Nordics. In: Bański J (ed) The Routledge Handbook of Small Towns.1st Edition Routledge, pp 218–231. 10.4324/9781003094203

[CR47] Steiger R (2011). The impact of snow scarcity on ski tourism: an analysis of the record warm season 2006/2007 in Tyrol (Austria). Tourism Review.

[CR48] Steiger R, Scott D, Abegg B, Pons M, Aall C (2019). A critical review of climate change risk for ski tourism. Curr Issue Tour.

[CR49] Steiger R, Knowles N, Pöll K, Rutty M (2022) Impacts of climate change on mountain tourism: a review. J Sustain Tour. In press. 10.1080/09669582.2022.2112204

[CR50] Stricker L (2022). Restricting the construction of second homes in tourist destinations: an effective intervention towards sustainability?. Swiss J Econ Stat.

[CR51] Töglhofer C, Eigner F, Prettenthaler F (2011). Impacts of snow conditions on tourism demand in Austrian ski areas. Climate Res.

[CR52] Vega H, Elhorst P (2015). The SLX model. J Reg Sci.

[CR53] Velvin J, Kvikstad TM, Drag E, Krogh E (2013). The impact of second home tourism on local economic development in rural areas in Norway. Tour Econ.

[CR54] Welling JT, Árnason Þ, Ólafsdottír R (2015). Glacier tourism: a scoping review. Tour Geogr.

[CR55] Willibald F, van Strien MJ, Blanco V, Grêt-Regamey A (2019). Predicting outdoor recreation demand on a national scale–the case of Switzerland. Appl Geogr.

[CR56] Willibald F, Kotlarski S, Ebner P, Bavay M, Marty C, Trentini FV, Ludwig R, Grêt-Regamey A (2021). Vulnerability of ski tourism towards internal climate variability and climate change in the Swiss Alps. Sci Total Environ.

[CR57] Wooldridge JM (1999). Distribution-free estimation of some nonlinear panel data models. J Econ.

[CR58] Yuheng L, Detotto C, Prunetti D, Stein A (2022). Unveiling spatial and temporal patterns of second home dynamics: a Bayesian spatiotemporal analysis for a Mediterranean island. Spat Econ Anal.

[CR59] Zhu X, Lee SY, Wen X, Wei Z, Ji Z, Zheng Z, Dong W (2021). Historical evolution and future trend of Northern Hemisphere snow cover in CMIP5 and CMIP6 models. Environ Res Lett.

[CR60] Zoğal V, Domènech A, Emekli G (2022). Stay at (which) home: second homes during and after the COVID-19 pandemic. J Tour Futur.

